# Associations of the oral microbiota and *Candida* with taste, smell, appetite and undernutrition in older adults

**DOI:** 10.1038/s41598-021-02558-8

**Published:** 2021-12-01

**Authors:** Kristina S. Fluitman, Tim J. van den Broek, Max Nieuwdorp, Marjolein Visser, Richard G. IJzerman, Bart J. F. Keijser

**Affiliations:** 1 Department of Internal Medicine, Amsterdam University Medical Center, Location VUmc, Amsterdam, The Netherlands; 2 Wallenburg Laboratory, Department of Molecular and Clinical Medicine, Sahlgrenska Academy, University of Gothenburg, Gothenburg, Sweden; 3 Amsterdam Public Health Research Institute, Amsterdam, The Netherlands; 4 Department of Microbiology and Systems Biology, TNO Healthy Living, Zeist, The Netherlands; 5 Department of Vascular Medicine, Amsterdam University Medical Center, Location AMC, Amsterdam, The Netherlands; 6 Department of Health Sciences, Faculty of Science, Vrije Universiteit Amsterdam, Amsterdam, The Netherlands; 7 Department of Preventive Dentistry, Academic Center for Dentistry Amsterdam, University of Amsterdam and Vrije Universiteit Amsterdam, Amsterdam, The Netherlands

**Keywords:** Next-generation sequencing, Epidemiology, Malnutrition, Oral diseases, Geriatrics, Microbial ecology

## Abstract

Poor taste and smell function are widely thought to contribute to the development of poor appetite and undernutrition in older adults. It has been hypothesized that the oral microbiota play a role as well, but evidence is scarce. In a cross-sectional cohort of 356 older adults, we performed taste and smell tests, collected anthropometric measurements and tongue swabs for analysis of microbial composition (16S rRNA sequencing) and *Candida albicans* abundance (qPCR). Older age, edentation, poor smell and poor appetite were associated with lower alpha diversity and explained a significant amount of beta diversity. Moreover, a lower *Streptococcus salivarius* abundance was associated with poor smell identification score, whereas high *C. albicans* abundance seemed to be associated with poor smell discrimination score. In our population, neither the tongue microbiota, nor *C. albicans* were associated with poor taste or directly with undernutrition. Our findings do suggest a host-microbe interaction with regard to smell perception and appetite.

## Introduction

Older adults over 65 years old make up the fastest growing age group worldwide. Currently, they comprise 20% of the European population^1^. These older adults are particularly prone to the development of undernutrition, which affects 9% of the community-dwelling and 28% of the institutionalized older adults^2^. Undernutrition is associated with various adverse events^3,4^ and is predominantly caused by a phenomenon coined “Anorexia of Aging”^5^. This is the reduction in appetite and energy intake that is often observed at older age. Factors such as cognitive impairments, depression, and polypharmacy, as well as poor taste and smell, poor dentition, and poor oral health are all considered to contribute to Anorexia of Aging^6,7^. It can be hypothesized that the oral microbiota is a new important factor in the Anorexia of Aging phenomenon, either by influencing taste or smell function^8–11^, or by other—as of yet unknown—mechanisms.

The oral microbiota constitute a complex, highly diverse microbiological community in the oral cavity^12^. Due to the anatomical proximity of the oral microbiota to taste and smell receptors, the two entities are thought to influence each other in several ways^13^. First, the oral microbiota metabolize and synthesize sensory active substances, affecting taste and smell receptor signaling in a process called “sensory adaption”^13^. Second, the oral microbiota may interact with the oral microenvironment, which in turn affects taste and smell function^9^. Finally, it was shown that germfree mice (lacking oral bacteria) developed altered olfactory epithelium compared to conventional mice and had a different olfactory neuronal response to olfactory stimuli^14^. Some studies have evaluated the association of the oral microbiota with taste function in humans. However, these studies reported inconsistent results, were limited in sample size, and did not focus specifically on older adults^8–11^. To our knowledge, we are the first to study the association of the oral microbiota with taste, smell, appetite and undernutrition in older adults.

In addition to the oral microbiota, the oral mycobiota—particularly *Candida—*may be associated with undernutrition. In a study of 218 young adults, candidiasis patients and healthy carriers of *Candida albicans* had significantly higher taste thresholds and were more likely to suffer from taste disorders than non-carriers. Oral candidiasis was also found to be associated with undernutrition^7^ and lower energy and protein intake^15^ in hospitalized older adults. Preliminary observational data suggested that the taste disorders^16^ and energy intake^15^ improved after treatment with antimycotica. Whether oral *C. albicans* is associated with poor smell, poor appetite or nutritional status at sub-pathological abundances is not yet known.

In this study, we aim to evaluate the oral microbiota and explore *C. albicans* from the tongue dorsum of 356 Dutch community-dwelling older adults in relation to taste and smell function, as well as poor appetite and undernutrition. A deeper understanding of the oral microbiota in relation to taste, smell, and appetite may further illuminate the complex pathophysiology of undernutrition in older adults and could possibly allow for the identification of preventative or therapeutic targets.

## Results

### Participant characteristics

A total of 356 well-phenotyped Dutch community-dwelling older adults from the ongoing Longitudinal Aging Study Amsterdam (LASA)^17–19^ were included in this cross-sectional cohort study. On average, participants were 73 years old (ranging from 65–93) and 207 (58.1%) were male. Undernutrition occurred in 76 (21.3%) participants, either based on low BMI (< 20 kg/m^2^ if < 70 years, or < 22 kg/m^2^ if ≥ 70 years)^20^ (n = 39) or on > 5% body weight loss averaged over the last 2 years (n = 43), or both. Poor appetite occurred in 20 (5.6%) participants based on a Council of Nutrition Appetite Questionnaire (CNAQ) score < 28^21^. Measured poor smell occurred in 16.5%, 22.0%, 19.7%, and 18.0% of participants (poor total smell score, poor smell threshold score, poor smell discrimination score, and poor smell identification score, respectively). Poor sour, salty, and umami taste scores were observed most often (31.5%, 37.7%, and 32.4%, respectively), whereas poor Bitter, sweet, and total taste scores were less common (7.3%, 15.5%, and 9.3%, respectively). An overview of all participant characteristics is shown in Supplementary Table S1.

### Associations of the tongue microbiota with taste, smell, appetite, and undernutrition

Microbiota composition was assessed by 16S rRNA sequencing of tongue swabs. Median sequencing depth was 94,703 reads/sample. In our cohort, 5367 amplicon sequencing variants (ASVs) were identified. Of these ASVs, 4429 were matched to known bacterial species from the Human Oral Microbiome Database (HOMD) with a sequence identity match > 90%^22^. The other 938 ASVs were minority members with a mean relative abundance of 0.000126% ± 0.001167. When filtering the 16S data to include only those ASVs contributing to the first 97.5% of all count data, 386 ASVs with an abundance relative to all count data of ≥ 0.01% each remained. These ASVs were matched to the HOMD with a mean 99.5% sequence identity match. The abundance filter was applied prior to all bio-statistical analysis, except for those concerning alpha-diversity. Sequencing of ten samples yielded less than 30.000 raw sequence reads and were excluded from further analysis.

First, the overall microbial composition was evaluated using alpha- and beta-diversity measures.

**Figure 1:**
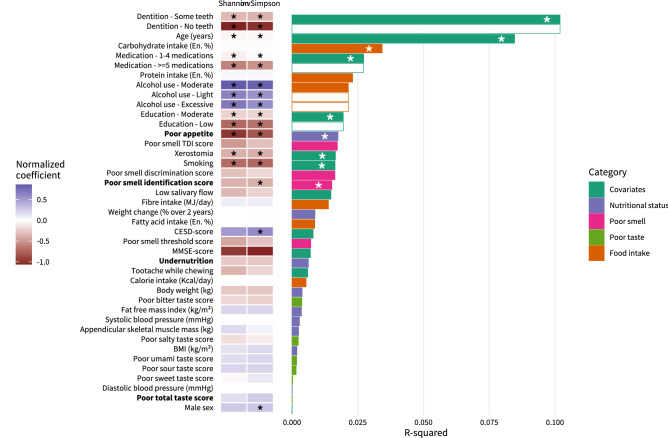
Association of clinical variables with microbiota alpha-diversity and beta-diversity. The heatmap depicts the normalized linear regression coefficients for the associations of clinical variables with Shannon and Inverse Simpson alpha-diversity. Blue indicates a positive association and red a negative association. Bar graphs depict the amount of variance explained by each clinical variable in the Bray–Curtis beta-diversity measure based on PERMANOVA models. Bars are colored based on the type of variable. White bars are shown for values of the same categorical variable. The variables poor appetite, poor smell identification, undernutrition, and poor total taste score concern our main hypotheses and are in written bold text. Asterisks indicate statistical significance at a *p* value < 0.01. *TDI score* Threshold discrimination identification score, *CESD* Centre of epidemiological studies depression scale, *MMSE* Mini-mental state exam, *BMI* Body mass index.

Alpha-diversity is an ecological measure used to indicate the intra-individual microbial diversity (i.e. how diverse is a participant’s oral microbiota). It was calculated using Shannon and inverse Simpson indices. Beta-diversity is used to indicate inter-individual dissimilarity in microbiota composition (i.e. how much does the overall microbiota composition differ between participants). It was calculated using the Bray–Curtis dissimilarity measure. We explored the univariate associations of all measured clinical variables with alpha- and beta-diversity (Fig. 1). The heatmap in Fig. 1 depicts the extent to which the clinical variables were associated with Shannon and Inverse Simpson alpha-diversity indices, analyzed with linear regression. Poor smell identification was significantly associated with lower diversity (*p* = 0.009 for Inverse Simpson), but none of the other smell or taste scores were. Poor appetite was also associated with lower alpha-diversity (*p* < 0.001 for Shannon and Inverse Simpson), but undernutrition, bodyweight, BMI, weight change, Fat Free Mass Index (FFMI), and Appendicular Skeletal Muscle Mass (ASMM) were not. Of the remaining variables, alcohol use, higher Centre for Epidemiologic Studies Depression (CESD)-score (indicating less depressive symptoms), and male sex were significantly associated with higher alpha-diversity. Having only some or no teeth, older age, more medication use, low or moderate education, xerostomia, and smoking were all significantly associated with lower alpha-diversity. The bar graphs in Fig. 1 depict the extent to which the clinical variables explained the variance in Bray–Curtis dissimilarity, analyzed with PERMANOVA analysis. Of all clinical variables, dentition explained the most variance in tongue microbial composition (R^2^ = 0.10, *p* < 0.001), followed by age (R^2^ = 0.08, *p* < 0.001). Of the smell scores, again only poor smell identification score explained a significant amount of variance (R^2^ = 0.02, *p* = 0.005). So did poor appetite (R^2^ = 0.02, *p* = 0.003), but none of the taste scores did, nor did undernutrition, weight, weight change, BMI, FFMI, or ASMM. Of the other variables, carbohydrate intake, medication use, education, xerostomia, and smoking explained significant amounts of variance. The precise coefficients of the analyses depicted in Fig. 1 are reported in Supplementary Table S2.

**Figure 2:**
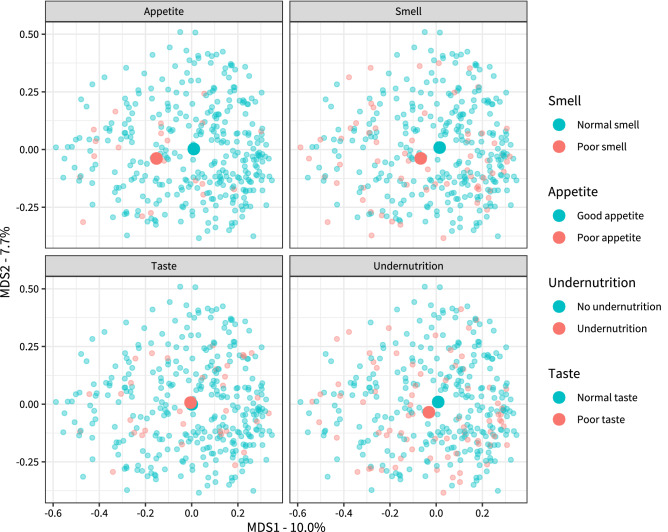
Ordination plots for Bray–Curtis dissimilarity colored for poor taste, smell, appetite, and undernutrition. Multi-dimensional scaling (or principal coordinate analysis) of the Bray–Curtis beta-dissimilarity in microbiota data. Each dot represents the microbiota sample of a single participant. Distance between samples represents the dissimilarity of these samples from each other. The axes represent 10.0% and 7.65% of variation in Bray–Curtis Distance. Samples are colored according to appetite, smell, taste, and undernutrition. *MDS* Multi-dimensional scaling.

**Figure 3:**
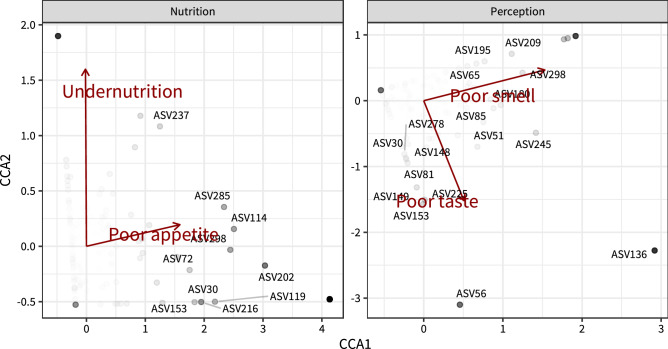
Canonical correspondence analysis of microbiota with poor taste, smell, appetite, and undernutrition. Canonical correspondence analysis depicting the species that most drive the correlations of the microbiota with poor appetite and undernutrition, or poor taste and poor smell. Poor appetite and poor smell identification are significantly associated with the microbiota data (CCA *p* = 0.010 and *p* = 0.001, respectively). Species are incorporated in the plot according to the interquartile rule, i.e. if their distance from the null-point (0,0) is greater than (1.5·IQR) + Q3 (in which IQR = Interquartile Range and Q3 = the third quartile). Species names corresponding to ASV numbers can be found in Supplementary Table S3. *CCA* Canonical correspondence analysis, *ASV* Amplicon sequence variance.

The Bray–Curtis dissimilarity is further visualized in Fig. 2, depicting a multi-dimensional scaling plot, in which each point represents the microbial composition of an individual participant. The further two points are apart, the more they differ based on Bray–Curtis dissimilarity. Samples are colored based on appetite, smell identification score, total taste score, or undernutrition. As was shown in Fig. 1, Bray Curtis dissimilarity is greater between participants with and without poor appetite or poor smell than between participants with and without poor taste or undernutrition. Next, we performed canonical correspondence analysis (CCA). This is a multivariate statistics technique in which correlations between two datasets are analyzed. Here, poor total taste score and poor smell identification score, or poor appetite and undernutrition make up one dataset, whereas the tongue microbiota (i.e. the abundance of all individual ASVs) make up the other. Again both poor appetite (*p* = 0.001) and poor smell identification (*p* = 0.010) were significantly correlated with the microbiota data, whereas poor taste and undernutrition were not. This is depicted in Fig. 3, as well as the bacterial species driving these correlations. Detailed results of the CCA analyses can be found in Supplementary Table S3.

**Figure 4:**
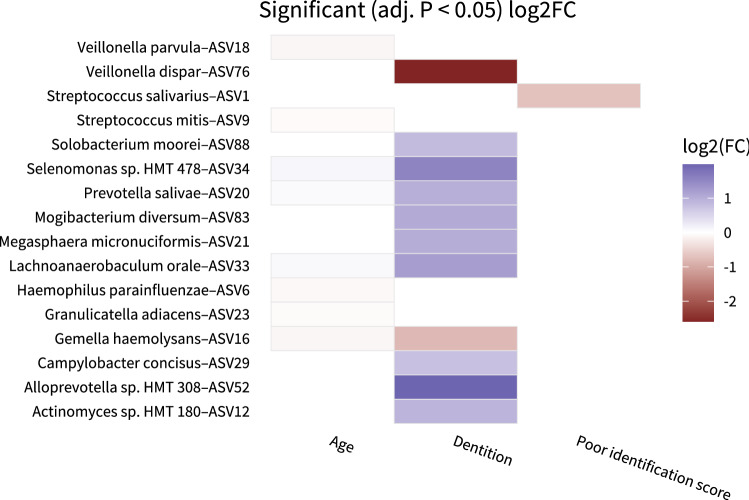
Microbiota taxa multivariately associated with age, dentition, poor taste, smell, appetite, and undernutrition outcomes. Heatmap depicting log2 fold change for taxa that are significantly (Benjamini–Hochberg adjusted *p* value < 0.05) associated with age, dentition, all poor smell scores, all poor taste scores, poor appetite, and undernutrition based on DESeq models. The association with age is adjusted for dentition, the association with dentition is adjusted for age, and the associations with all poor taste scores, all poor smell scores, poor appetite and undernutrition are adjusted for both age and dentition. Blue indicates taxa that increase in abundance with these conditions and red indicates taxa that decrease in abundance.

Finally, we examined the associations of individual microbial taxa with poor smell, taste, appetite, and undernutrition using DESeq analyses. In our univariate analyses, we identified various taxa that were differentially abundant for our clinical outcomes. These results are depicted in Supplementary Fig. 1 and Supplementary Table S4. Because our previous analyses (Fig. 1) identified age and dentition as most important microbiota determinants, we adjusted our DESeq analyses for both age and dentition (Fig. 4 and Supplementary Table S5). Then only the association of a lower abundance of *Streptococcus salivarius* with poor smell identification score remained (log2 Fold Change = -0.69, adjusted *p* = 0.003). No other taxa were significantly differentially abundant for any of the other taste or smell scores, nor for appetite or undernutrition. Altogether, we demonstrated that both poor appetite and poor smell function are univariately associated with the overall tongue microbiota composition (alpha- and beta-diversity) in older adults. We also showed that participants with poor smell identification have a lower abundance of *S. salivarius* after adjustment for age and dentition.

### Associations of *Candida albicans* with taste, smell, appetite, and undernutrition

The abundance of *C. albicans* was determined with qPCR of the tongue swabs. A cross-table with the proportions of participant with no *C. albicans*, low *C. albicans* abundance, and high *C. albicans* abundance that have poor taste, smell, appetite or undernutrition can be found in Supplementary Table S6. With logistic regression analyses (both crude and adjusted), we found *C. albicans* carriers (low or high abundance versus no *C. albicans present*) did not have a higher risk of poor taste, poor appetite, or undernutrition (Table 1). However, in our population, participants with high abundance of *C. albicans* did have 4.7 times higher odds for poor smell discrimination than participants with no *C. albicans,* after adjustment for age, dentition, sex, educational status, smoking status, medication use, CESD-score, MMSE-score (Bonferroni adjusted *p* value: 0.048).

**Table 1:** Logistic regression of low or high *Candida albicans *abundance versus no *C. albicans* with poor taste, smell, appetite, and undernutrition.

	Low versus no *C. albicans*		High versus no *C. albicans*	
	OR (95%-CI)	*p* value	OR (95%-CI)	*p* value
**Poor total taste**				
Crude	1.02 (0.40–2.58)	0.965	1.52 (0.66–3.51)	0.327
Adjusted	1.01 (0.39–2.66)	0.972	1.45 (0.56–3.74)	0.447
**Poor sweet taste**				
Crude	0.74 (0.37–1.47)	0.384	0.87 (0.43–1.79)	0.713
Adjusted	0.73 (0.35–1.49)	0.384	0.92 (0.40–2.07)	0.831
**Poor salty taste**				
Crude	0.81 (0.47–1.38)	0.430	0.63 (0.37–1.06)	0.084
Adjusted	0.85 (0.49–1.48)	0.562	0.66 (0.36–1.22)	0.187
**Poor sour taste**				
Crude	0.99 (0.57–1.72)	0.962	0.99 (0.57–1.72)	0.962
Adjusted	1.00 (0.56–1.76)	0.987	1.19 (0.63–2.26)	0.591
**Poor bitter taste**				
Crude	0.85 (0.31–2.35)	0.752	0.62 (0.24–1.58)	0.316
Adjusted	0.81 (0.28–2.36)	0.701	1.10 (0.35–3.39)	0.875
**Poor umami taste**				
Crude	1.07 (0.61–1.87)	0.813	0.86 (0.50–1.48)	0.581
Adjusted	1.12 (0.62–2.03)	0.708	1.25 (0.66–2.39)	0.495
**Poor smell TDI**				
Crude	0.85 (0.24–3.01)	0.804	1.00 (0.37–2.69)	0.996
Adjusted	0.79 (0.18–3.47)	0.755	0.55 (0.16–1.93)	0.349
**Poor smell threshold**				
Crude	0.92 (0.33–2.57)	0.868	0.49 (0.19–1.26)	0.138
Adjusted	0.55 (0.16–1.89)	0.342	0.28 (0.09–0.92)	0.036
**Poor smell discrimination**				
Crude	2.59 (0.68–9.83)	0.162	5.86 (2.00–17.20)	0.001*
Adjusted	2.45 (0.58–10.40)	0.225	4.70 (1.40–15.75)	0.012*
**Poor smell identification**				
Crude	1.66 (0.85–3.26)	0.141	2.19 (1.15–4.17)	0.018
Adjusted	1.67 (0.83–3.37)	0.152	1.44 (0.67–3.06)	0.349
**Poor appetite**				
Crude	0.20 (0.03–1.59)	0.129	1.77 (0.68–4.58)	0.239
Adjusted	0.21 (0.03–1.82)	0.157	0.63 (0.15–2.67)	0.527
**Undernutrition**				
Crude	0.85 (0.44–1.65)	0.631	1.31 (0.71–2.40)	0.385
Adjusted	0.85 (0.42–1.72)	0.660	1.09 (0.52–2.27)	0.828

Shown are odds ratio (OR), 95% confidence interval (95%-CI), and uncorrected *p* values. Asterisks indicate statistical significance (*p* < 0.008 for poor taste; *p* < 0.013 for poor smell (Bonferroni corrected); or *p* < 0.05 for poor appetite or undernutrition). Adjusted models include covariates: age, dentition, sex, educational status, smoking status, medication use, CESD-score, MMSE-score. Poor taste: total taste score < 6, sweet score < 2, sour score < 2, salty score < 2, bitter score < 1, umami score < 1. Poor smell: TDI-score ≤ 19.5, T-score ≤ 2.5, D-score ≤ 7, I-score ≤ 9. Poor appetite: Council of Nutrition Appetite Questionnaire score < 28. Undernutrition: > 5% bodyweight loss averaged over 2 years or BMI < 20 (if age < 70) or BMI < 22 (if age > 70) or both.

## Discussion

In this study, we aimed to evaluate the tongue microbiota and explore *C. albicans* in relation to poor appetite and undernutrition in older adults. In our sample of Dutch community-dwelling older adults, alpha- and beta-diversity of the tongue microbiota were associated with poor smell and poor appetite, but not with poor taste or undernutrition. Moreover, a lower abundance of *S. salivarius* was significantly associated with poor smell identification and a high abundance of *C. albicans* seemed to be associated with poor smell discrimination.

To our knowledge, we are the first to study the associations of the oral microbiota with olfactory function in humans. Lower intra-individual (alpha) diversity was associated with poor smell identification, which also explained a significant amount of variance in inter-individual (beta) diversity, measured by Bray–Curtis dissimilarity. CCA analysis confirmed the association of oral microbiota with poor smell identification. Moreover, when studying the associations with individual bacterial taxa, a lower abundance of *S. salivarius* was associated with poor smell identification, even after adjustment for both age and dentition. *S. salivarius* is a prominent member of the tongue microbiota. It is a facultative anaerobic bacterium and is considered to be one of the first colonizers of the human oral cavity at birth^23^. Both in our study population and in a previous study of older adults, it was the single most abundant taxon on the tongue dorsum^24^. *S. salivarius* has been extensively studied for its anti-inflammatory properties and possible application as oral probiotic^25^. Live *S. salivarius* were shown to interfere with virulent respiratory pathogens such as *Streptococcus mutans, Streptococcus sobrinus,* and especially *Streptococcus pyogenes* which is typically involved in causing pharyngitis^23,26,27^. Possibly, a lower abundance of *S. salivarius* increases the risk of nasopharyngeal infections and inflammation, contributing to inflammation-associated olfactory dysfunction^28^. Our results suggest *S. salivarius*-based probiotic therapy may benefit older adults with hyposmia, although our findings need to be replicated and causality must be established first.

The association of oral microbiota with taste function has never been studied in a cohort as large as ours, nor specifically in older adults^8–11^. Contrary to poor smell, but consistent with previous studies^8,10^, neither alpha-diversity, nor beta-diversity were associated with poor taste. Cattaneo et al.^10^ attributed the lack of associations with alpha- or beta-diversity in part to the limited inter-personal variation in oral microbiota compared to other body sites. However, in our sample of older adults we did find differences in both alpha- and beta- diversity for outcomes other than poor taste. As did the previous studies^8–11^, we identified several differentially abundant taxa for taste in our univariate DESeq analysis. However, unlike these studies, we adjusted our analysis for age and dentition. This rendered the associations with individual taxa non-significant. It is possible that several of the associations found in the previous studies would disappear when adjusting for age or oral health status.

Associations between the oral microbiota and poor oral health^24^, and between poor oral health and poor appetite^6^ have been established in literature. However, the direct association of the oral microbiota with poor appetite has not been studied before. In our sample, lower alpha-diversity was associated with poor appetite. Poor appetite also explained a significant amount of variance in beta-diversity and was significantly associated with the microbiota as confirmed by our CCA analysis. Moreover, poor appetite was associated with a significant log2 fold change in numerous bacterial taxa, but these associations did not hold after adjustment for age and dentition. This suggests that both the oral microbiota and appetite are associated with older age and/or dentition. Since we did find several significant associations before adjustment of the analysis, an indirect relation of oral microbiota with appetite confounded by oral health (or edentation) is possible, but a direct association is less likely.

Although we found associations of alpha- and beta-diversity with poor smell and poor appetite, we found no associations with undernutrition, nor with BMI, previous weight change, FFMI, or ASMM. This could be due to the relatively vital, community-dwelling older population we studied. Undernutrition in community-dwelling older adults is typically less prevalent or pronounced than in less resilient and institutionalized older adults^29^. Moreover, undernutrition is a complex, multi-factorial condition^6^ that may only be expressed when sufficient contributing factors are present. Even if exposed to less desirable, so-called “dysbiotic” oral microbiota, our population may be resilient from developing undernutrition due to sufficient protective circumstances. Possibly, only when enough factors predispose an individual to undernutrition, does the oral microbiota play an active contributing role in its etiology. This would result in a notion of opportunistic dysbiotic microbiota, similar to the way in which an opportunistic bacterium only causes disease under specific circumstances. Future studies, in more sub-populations, are needed to identify what characterizes dysbiotic oral microbiota in regard to undernutrition.

In addition to the tongue microbiota, we explored the relation of *C. albicans* with poor taste, smell, appetite, and undernutrition. *Candida* and candidiasis have previously been suggested to affect both undernutrition^7,15^ and taste function^16^. Contrary to the study by Sakashita et al.^16^, high *C. albicans* abundance was not associated with poor taste in our population. The discrepancy between studies may be due to differences in analysis techniques or study population. Whereas we determined *C. albicans* abundance by qPCR and studied older adults, Sakashita, et al. determined *C. albicans* presence by microscopic examination of cultured tongue scrapings in young adults. To our knowledge we are the first to evaluate the association of oral *Candida* with poor smell. In our exploratory study we found some evidence that high *C. albicans* abundance is associated with poor discriminatory smell function (borderline significant). However, we found no associations with the total, threshold, or identification smell scores. Possibly the association of *C. albicans* with a diminished ability to discriminate between odors is due to higher levels of inflammation in the nasopharynx. More dedicated research is needed to confirm the relationship between *C. albicans* and smell function.

Some strengths and limitations to this study are worth mentioning. First, in comparison to previous studies we included a large population^8–11^. We also evaluated the full spectrum of the tongue microbiota using 16S rRNA sequencing, rather than depending on targeted culture-dependent techniques^9^. Third, we corrected our final DESeq analysis for multiple testing and adjusted for possible confounding by age and dentition, which appear to be the most important microbiota determinants based on our results. However, the possibility of residual confounding remains. Finally, we considered associations with both overall community structure (alpha- and beta-diversity measures) and individual species. A potential limitation of this study is its cross-sectional design, which keeps us from evaluating causality. Due to complex host-microbe interactions bi-directionality of many associations seems likely. However, as little to no research has yet been done on the topic, establishing cross-sectional associations serves as an important first explorative step. We did not conduct full metagenomics, nor could we assess the functional capacity of the oral microbiome. Possibly, there was no association with the microbial community in some instances, but there would be with bacterial metabolic pathways. Finally, we assessed the oral microbiota on the posterior tongue dorsum, which is only one of many bacterial niches in the oral cavity^22^, although we believe it to be the most suitable for our study as the tongue dorsum is a major microbial reservoir due to its papillary structure with bacterial loads increasing from anterior to posterior^8^. Additionally, the tongue microbiota are most approximate to the taste receptors.

In conclusion, we found several associations of the tongue microbiota with poor smell and poor appetite, but not with poor taste or undernutrition. Specifically, a lower abundance of *S. salivarius* was associated with poor smell, even after adjustment for age or dentition. These results need to be further explored in future studies, but do suggest a host-microbe interaction with regard to smell function and appetite.

## Methods

### Participants

This concerns a cross-sectional ancillary-study of the ongoing LASA-study^17–19,30^. LASA participants with available body measurements from the 2015/16 LASA data-collection wave (n = 1642) were pre-screened for inclusion based on LASA data. Eligible participants (n = 727) were then contacted by phone and screened for the remaining exclusion criteria. Exclusion criteria were: age < 65 years, active institutionalization or > 4 weeks institutionalization in the three months prior to this study, over-nutrition established either by BMI > 30 kg/m^2^ on last LASA measurement (2015/16) or > 2% body weight gain between latest two LASA measurements (2011/13 to 2015/16), diagnosed active malignancy, systemic antibiotics, immune suppression, and MMSE-score < 18. Informed consent and all current measurements were obtained during a single home visit (n = 360), in 2017/18. In 1 participant no accurate body measurements could be obtained and in 3 participants no tongue swab could be obtained, leaving 356 participants for the statistical analyses. This study was approved by the local medical ethics committee (Medisch Ethische Toetsingscommissie VUmc, The Netherlands) and carried out in accordance with the Declaration of Helsinki.

### Anthropometrics

Participants wore only undergarments when measuring body weight. Some wore indoors clothing without shoes, in which case 1 kg was subtracted^31^. Weight change was calculated in % bodyweight averaged over the last 2 years. Undernutrition was defined either by low BMI (BMI < 20 kg/m^2^ if age < 70 years or BMI < 22 kg/m^2^ if age ≥ 70 years)^20^, or by more than 5% bodyweight loss over 2 years. Body impedance analysis was performed using the BodyStat 1500MDD device (Bodystat Ltd., Isle of Man, UK). FFMI and ASMM were predicted using the validated formulas from Kyle et al.^32^ and Sergi et al.^33^, respectively^34^. Blood Pressure was measured at the left arm in duplicate using an automatic Omron M7 device (Omron Corporation, Tokyo, Japan).

### Appetite, oral health, food intake, and covariates

Poor appetite was defined as CNAQ-score < 28^21^. Oral health was evaluated using a questionnaire previously used in LASA^35^. Dentition was categorized as follows: no original teeth left (“no teeth”), ≤ 7 teeth in either upper or lower jaw left (“some teeth), or > 7 teeth in both the upper and lower jaw (“most-all teeth”). The 238-item Dutch version of the Food Frequency Questionnaire (FFQ) from the Healthy Life in an Urban Setting study was used to assess energy intake (kcal/d) and macronutrient intake (EN%)^36^. This FFQ was validated in a cohort of Dutch older adults^37^. Data on socio-demographics (age, sex, educational status), lifestyle factors (Alcohol use^38^ and smoking status), co-morbidities (CES-D depression scores^39^, medication use), and cognitive status (MMSE-score^40^) were previously collected by LASA. Education was categorized as low (elementary education or lower), moderate (lower vocational education through general secondary education), and high (higher vocational education through university education). Alcohol use was categorized as none, light, moderate, or excessive based on the Garretsen alcohol consumption index^38^. Medication use was categorized as no medication, 1–4 medicines, > 4 medicines.

### Sensory testing

Taste function was assessed using commercially available taste strips (Burghart Messtechnik GmbH, Wedel, Germany). The test consisted of 22 paper taste strips, 20 of which are impregnated with increasing concentrations of sweet, sour, salty, bitter, and umami tastes, 2 are tasteless^41^, reproducing the test validated by Mueller et al.^42^. Each individual taste score ranges from 0 to 4, and the total score ranges from 0 to 20. A total taste score < 6, sweet score < 2, sour score < 2, salty score < 2, bitter score < 1, or umami score < 1 was considered poor.

Smell function was measured with the commercially available Sniffin’ Sticks (Burghart Messtechnik GmbH, Wedel, Germany), consisting of an odor identification test (score 0–16), an odor discrimination test (score 0–16), and an odor threshold test (score 0–16), which sum up to form the total Threshold-Discrimination-Identification (TDI-) score^43^. An identification score ≤ 9, discrimination score ≤ 7, threshold score ≤ 2.5, or TDI-score ≤ 19.5 was considered poor^43^. Not all participants completed the threshold and discrimination tests, as they are time consuming, can be considered too burdensome, and not all researchers performing the home visits were equipped to carry them out. The smell identification test was performed in all visits and was therefore used as our overall measurement for olfactory function^44–49^. Whether the discrimination and threshold tests were performed was decided prior to the visit based on the participant’s preference and researcher available. Participants were asked not to smoke, chew gum, eat, or drink anything other than water in the hour preceding the sensory tests.

### Tongue swap

Participants were asked to stay fasted and not smoke or chew gum in the hour prior to tongue sampling, nor did they brush their teeth that day. Tongue swabs were collected in duplicate by stroking a Copan eNAT swab (Copan Italia S.p.A., Bréscia, Italy) over the posterior tongue dorsum for four times. The swab was then inserted in a tube containing the eNAT RNA/DNA stabilizing and preservation medium. It was chilled in an icepack until storage at -80 °C on the same day.

### 16S rRNA analysis

16S rDNA amplicon sequencing of the V4 hypervariable region was performed as described previously^50^. Each sample was PCR amplified using 1 ng of template DNA with primers F515/R806, targeting the V4 hypervariable region of the 16 s ribosomal gene^51^. In each batch of samples, a mixed pure culture isolates (mock), blanco extraction controls, pooled salivary extraction controls and pooled DNA amplification controls were included. The amount of DNA per sample was quantified using the Quant-iT™ PicoGreen® dsDNA Assay Kit (Thermo Fisher Scientific). The amplicon libraries were pooled in equimolar amounts and purified using the IllustraTM GFXTM PCR DNA and Gel Band Purification Kit (GE Healthcare, Eindhoven, The Netherlands). Amplicon quality and size was analyzed on the Fragment Analyzer (Advanced Analytical). Paired-end sequencing of amplicons was conducted in five separate runs on the Illumina MiSeq platform (Illumina, Eindhoven, The Netherlands). Denoising of sequence data and identification of ASVs were performed using the DADA2 (1.12.1) pipeline^52^.

### Candida qPCR

Quantitative assessment of *C. albicans* abundance was performed by quantitative PCR using primers listed in Supplementary Table S7^53^. qPCR was performed in RT PCR master mix (Diagenode, Seraing, B) on an Applied Biosystems 7500 RT PCR system during 40 cycles of with a denaturation step at 95.0 °C for 15 s and an annealing/elongation step at 60.0 °C for 1 min. A standard curve was established by analysis of genomic DNA of *C. albicans* ATCC 90,028. Cell densities were estimated by accounting for the elution volume used after DNA extraction (60 μl) and assuming a (diploid) genome size of 14 Mb for *C. albicans*. *C. albicans* abundance was then categorized into 3 groups: no *C. albicans* present, low *C. albicans* abundance (0 counts through the median number of counts), and high *C. albicans* abundance (the median number of counts through the highest number of counts).

### Statistical analysis

All statistical analyses of the microbiota were performed using R version 3.5.1^54^. All figures were composed using the *ggplot2* package version 3.2.1^55^. Multivariate analyses and ordinations were performed using the *vegan* package, version 2.5-3^56^. This package was also used to calculate the inverse Simpson and Shannon α-diversities. Associations of the clinical variables with the α-diversity measures were determined using univariate linear regression from the *base* R package. For all analyses, with the exception of those concerning α-diversity, the 16S data was filtered to include only those ASVs that contribute to the first 97.5% of all counts in the data. The R^2^ in Bray–Curtis β-diversity for the clinical variables was determined by fitting vectors for continuous variables and weighted centroids for categorical variables. Significance of these R^2^ values was calculated using PERMANOVA analysis. The metric multi-dimensional scaling ordination is a distance-based method; in this case Bray–Curtis distances were used. This is a non-Euclidean metric, therefore the reported explained variance for the first two axes are based on Cailliez-corrected eigenvalues^57^. Canonical correspondence analysis (CCA) is a method often used to identify correlations between two data matrixes, in this case between the microbiota data and taste, smell, appetite, and undernutrition. The multivariate models fitted by CCA were tested by permutation analysis in order to produce Type III (marginal) *p* values for the terms included in the model. 10 × 10^2^ permutations were used for all reported results. The species weights for this model are reported as weighted averages calculated from the microbiota composition and the model variables. Regression on the microbiota count data was in all cases performed using the *DESeq2* package, version 1.20.0^58^. Log2 fold change in microbiota count was calculated for age, dentition, poor smell, poor taste, poor appetite, and undernutrition univariately and adjusted for age, dentition, or age and dentition. *p* values were corrected for multiple testing according to the Benjamini–Hochberg procedure.

For the statistical analyses concerning *Candida* data, SPSS software version 22 (SPSS Inc., Chicago Illinois) was used. The associations of *C. albicans* (low abundance versus none, and high abundance versus none) with all taste and smell scores, poor appetite, and undernutrition were explored with logistic regression. These models were first run univariately and then adjusted for age, dentition, sex, educational status, smoking status, medication use, CESD-score, MMSE-score. As all individual taste scores make up the overall taste test and all individual smell scores make up the overall smell test, Bonferroni correction for multiple testing was carried out for models with taste or smell scores as outcome. Bonferroni corrected alpha-levels used for statistical significance were 0.008 (= 0.05/6) for taste and 0.013 (= 0.05/4) for smell, respectively. Bonferroni uncorrected *p* values are reported.

## Supplementary Information

Supplementary Information 1.

Supplementary Information 2.

Supplementary Information 3.

Supplementary Information 4.

## Data Availability

The 16S rRNA gene sequences that support the findings of this study have deposited in the restricted access data repository: the European Genome-Phenome Archive (https://ega-archive.org/), dataset ID: EGAD00001007705. The 16S rRNA gene sequences, as well as the associated metadata are available upon request from the Longitudinal Aging Study Amsterdam (https://lasa-vu.nl). The data are not publicly available due to ethical and privacy considerations.
