# Supplementary material for: Associations of the oral microbiota and Candida with taste, smell, appetite and undernutrition in older adults
Source: Sci Rep. 2021 Dec 1;11:23254. doi: 10.1038/s41598-021-02558-8 (PMC8636608; doi:10.1038/s41598-021-02558-8)
Supplement: Supplementary file 1 — Supplementary Information 1. [file 41598_2021_2558_MOESM1_ESM.docx]

**Associations of the oral microbiota and *Candida* with taste, smell, appetite and undernutrition in older adults**

Kristina S. Fluitman^1,2,3^ [k.fluitman@amsterdamumc.nl](mailto:k.fluitman@amsterdamumc.nl)

Tim van den Broek^4^ [tim.vandenbroek@tno.nl](mailto:tim.vandenbroek@tno.nl)

Max Nieuwdorp^1,5^ [m.nieuwdorp@amsterdamumc.nl](mailto:m.nieuwdorp@amsterdamumc.nl)

Marjolein Visser^3,6^ [m.visser@vu.nl](mailto:m.visser@vu.nl)

Richard G. IJzerman^1^ [rg.ijzerman@amsterdamumc.nl](mailto:rg.ijzerman@amsterdamumc.nl)

Bart J.F. Keijser^4,7^* [Bart.keijser@tno.nl](mailto:Bart.keijser@tno.nl)

^1^ Department of Internal Medicine, Amsterdam University Medical Centers, location VUmc, Amsterdam, The Netherlands

^2^ Wallenburg Laboratory, Department of Molecular and Clinical Medicine, Sahlgrenska Academy, University of Gothenburg, Gothenburg, Sweden

^3^ Amsterdam Public Health Research Institute, Amsterdam, The Netherlands

^4^ Department of Microbiology and Systems biology, TNO earth, Life and Social Sciences, Zeist, The Netherlands

^5^ Department of Vascular Medicine, Amsterdam University Medical Centers, Location AMC, Amsterdam, The Netherlands

^6^ Department of Health Sciences, Faculty of Science, Vrije Universiteit Amsterdam, Amsterdam, The Netherlands.

^7^ Department of Preventive Dentistry, Academic Center for Dentistry Amsterdam, University of Amsterdam and Vrije Universiteit Amsterdam, Amsterdam, The Netherlands

**Supplementary Table S1: Characteristics for all included LASA participants**

| **Variables** | **n** |  |
| --- | --- | --- |
| **Demographics** | | |
| Age (yrs) | 356 | 73 [69-77] |
| Sex (male) | 356 | 207 (58.1) |
| **Nutritional state** | | |
| Undernutrition^1^ | 356 | 76 (21.3) |
| Poor appetite (CNAQ score) | 355 | 31 [30-33] |
| Poor appetite^2^ |  | 20 (5.6) |
| BMI (kg/m2) | 356 | 24.8 ± 2.5 |
| Low BMI^3^ |  | 39 (21.3) |
| Weight difference (%bodyweight/2yrs) | 356 | -0.5 ± 4.5 |
| Weight loss^4^ |  | 43 (12.1) |
| FFMI (kg/m2) | 341 | 17.5 ± 2.1 |
| Low FFMI^5^ |  | 65 (19.2) |
| ASMM (kg) | 341 | 20.2 ± 3.9 |
| Low ASMM^6^ |  | 59 (17.3) |
| Systolic Blood Pressure (mmHg) | 355 | 138.9 ± 19.2 |
| Diastolic Blood Pressure (mmHg) | 355 | 81.2 ± 10.4 |
| **Food Frequency Questionnaire** | | |
| Calory intake (kcal/day) | 352 | 2118.2 ± 531.3 |
| Carbohydrate intake (En%) | 352 | 41.0 ± 6.6 |
| Protein intake ( En%) | 352 | 14.7 ± 2.2 |
| Fatty acid intake ( En%) | 352 | 34.9 ± 5.7 |
| Fibre intake (MJ) | 352 | 2.5 ± 0.6 |
| **Taste and Smell-scores** | | |
| Poor TDI-score^7^ | 139 | 23 (16.5) |
| Poor T-score^8^ | 141 | 31 (22.0) |
| Poor D-score^9^ | 142 | 28 (19.7) |
| Poor I-score^10^ | 355 | 64 (18.0) |
| Poor total taste-score^11^ | 355 | 33 (9.3) |
| Poor sweet^12^ |  | 55 (15.5) |
| poor sour^13^ |  | 112 (31.5) |
| poor salty^14^ |  | 134 (37.7) |
| Poor bitter^15^ |  | 26 (7.3) |
| Poor umami^16^ |  | 115 (32.4) |
| **Oral health** | | |
| Dentition | 356 |  |
| No teeth |  | 80 (22.5) |
| Some teeth |  | 79 (22.2) |
| Most-all teeth |  | 197 (55.3) |
| Toothache while chewing last 6 months | 355 | 18 (5.1) |
| Xerostomia last 6 months | 355 | 97 (27.3) |
| Salivary flow (g/min) | 147 | 0.9 [0.6-1.5] |
| **Covariates** | | |
| Alcohol | 356 |  |
| No alcohol use |  | 25 (7.0) |
| Light |  | 191 (53.7) |
| Moderate |  | 117 (32.9) |
| Excessive |  | 23 (6.5) |
| Current smoker | 356 | 26 (7.3) |
| MMSE-score | 356 | 29 [28-29] |
| CESD-score | 354 | 4 [2-8] |
| Medication | 356 |  |
| 0 drugs |  | 98 (27.5) |
| 1-4 drugs |  | 188 (52.8) |
| ≥ 5 drugs |  | 70 (19.7) |
| Education | 356 |  |
| Low |  | 39 (11.0) |
| Medium |  | 185 (52.0) |
| High |  | 132 (37.1) |

Data is depicted in mean ± standard deviation, median [interquartile range] and number (percentage).

CNAQ: Council of Nutrition Appetite Questionnaire; BMI: Body Mass Index; FFMI: Fat Free Mass Index; ASMM: Appendicular Skeletal Muscle Mass; Kcal: kilocalorie; En%: percentage energy intake; MJ: megajoule; TDI: Threshold Discrimination Identification; MMSE: Mini Mental State Exam; CESD: Center of Epidemiological Studies Depression.

1>5% bodyweight loss averaged over 2 years or BMI<20 (if age <70) or BMI<22 (if age >70); 2CNAQ-score <28; 3BMI < 20 (age <70) / < 22 (age >/= 70); 4Weight loss >5% bodyweight/2 years; 5FFMI < 17 (men) / < 15 (women); 6ASMM <21.4 (men) / < 14.1 (women); 7TDI-score ≤19.5; 8T-score ≤2.5; 9D-score ≤ 7; 10I-score ≤ 9; 11total taste score < 6; 12sweet score < 2; 13sour score < 2; 14salty score < 2; 15bitter score < 1; 16umami score < 1

**Supplementary Table S2: Clinical variables associated with oral microbiota alpha- and beta-diversity**

|  | **Shannon Index** | | **Inversed Simpson Index** | | **Bray-Curtis dissimilarity** | |
| --- | --- | --- | --- | --- | --- | --- |
| **variable** | **Normalized regression coefficient** | **p-value** | **Normalized regression coefficient** | **p-value** | **R^2^** | **p-value** |
| Dentition  Some teeth  No teeth | -0.32  -1.05 | 0.000  0.000 | -0.35  -1.00 | 0.000  0.000 | 0.102 | 0.000 |
| Age | -0.05 | 0.000 | -0.04 | 0.000 | 0.085 | 0.000 |
| Carbohydrate intake | -0.02 | 0.031 | -0.02 | 0.044 | 0.034 | 0.003 |
| Medication  5 or more medications  1 to 4 medications | -0.59  -0.07 | 0.000  0.000 | -0.52  -0.03 | 0.001  0.001 | 0.027 | 0.001 |
| Protein intake | 0.04 | 0.106 | 0.04 | 0.077 | 0.023 | 0.021 |
| Alcohol use  Moderate  Light  Very excessive | 0.86  0.63  0.69 | 0.002  0.002  0.002 | 0.82  0.56  0.57 | 0.002  0.002  0.002 | 0.021 | 0.019 |
| Education  Moderate  Low | -0.21  -0.72 | 0.000  0.000 | -0.19  -0.68 | 0.001  0.001 | 0.020 | 0.011 |
| Poor appetite | -0.97 | 0.000 | -0.81 | 0.000 | 0.017 | 0.002 |
| Poor TDI score | -0.48 | 0.054 | -0.29 | 0.197 | 0.017 | 0.099 |
| Xerostomia | -0.37 | 0.002 | -0.37 | 0.002 | 0.017 | 0.003 |
| Smoking | -0.71 | 0.000 | -0.73 | 0.000 | 0.016 | 0.003 |
| Poor discrimination score | -0.29 | 0.226 | -0.18 | 0.390 | 0.016 | 0.110 |
| Poor identification score | -0.36 | 0.010 | -0.36 | 0.009 | 0.015 | 0.005 |
| Salivary flow | -0.34 | 0.072 | -0.21 | 0.212 | 0.015 | 0.122 |
| Fibre intake | 0.09 | 0.323 | 0.09 | 0.335 | 0.014 | 0.089 |
| Weight change | 0.00 | 0.905 | 0.00 | 0.834 | 0.009 | 0.225 |
| Fatty acid intake | 0.00 | 0.871 | 0.00 | 0.606 | 0.009 | 0.218 |
| CESD | 0.51 | 0.022 | 0.58 | 0.010 | 0.008 | 0.066 |
| Poor threshold score | -0.42 | 0.066 | -0.28 | 0.168 | 0.007 | 0.362 |
| MMSE | -0.96 | 0.176 | -1.05 | 0.140 | 0.007 | 0.073 |
| Undernutrition | -0.25 | 0.056 | -0.25 | 0.050 | 0.006 | 0.108 |
| Tootache w/ chewing | -0.35 | 0.149 | -0.20 | 0.414 | 0.006 | 0.128 |
| Calorie intake | 0.00 | 0.384 | 0.00 | 0.430 | 0.005 | 0.396 |
| Weight | -0.26 | 0.112 | -0.26 | 0.116 | 0.004 | 0.268 |
| Poor bitter taste | -0.19 | 0.361 | -0.21 | 0.300 | 0.004 | 0.272 |
| Fat Free Mass Index | 0.20 | 0.145 | 0.21 | 0.135 | 0.004 | 0.301 |
| Systolic blood pressure | 0.00 | 0.766 | 0.00 | 0.531 | 0.003 | 0.617 |
| Appendicular Skeletal Muscle Mass | 0.18 | 0.226 | 0.06 | 0.672 | 0.002 | 0.437 |
| Poor salty taste | -0.15 | 0.176 | -0.09 | 0.428 | 0.002 | 0.423 |
| BMI | 0.15 | 0.386 | 0.18 | 0.301 | 0.002 | 0.525 |
| Poor umami taste | 0.17 | 0.134 | 0.20 | 0.089 | 0.002 | 0.542 |
| Poor sour taste | 0.20 | 0.081 | 0.21 | 0.064 | 0.002 | 0.573 |
| Poor sweet taste | -0.03 | 0.865 | 0.11 | 0.462 | 0.000 | 0.922 |
| Diastolic blood pressure | 0.00 | 0.711 | 0.00 | 0.960 | 0.000 | 0.982 |
| Poor total taste score | 0.17 | 0.368 | 0.26 | 0.163 | 0.000 | 0.971 |
| Male sex | 0.27 | 0.014 | 0.28 | 0.010 | 0.000 | 0.981 |

Shown are the normalized regression coefficients and p-values for linear regression models of all clinical variables with Shannon and Inverse Simpson alpha-diversity indices, as well as the R^2^ (variance explained) in Bray-Curtis beta-diversity by each clinical variable and corresponding p-values based on PERMANOVA models. TDI score: Threshold Discrimination Identification score; CESD: Centre of Epidemiological Studies Depression scale; MMSE: Mini-Mental State Exam; BMI: Body Mass Index.


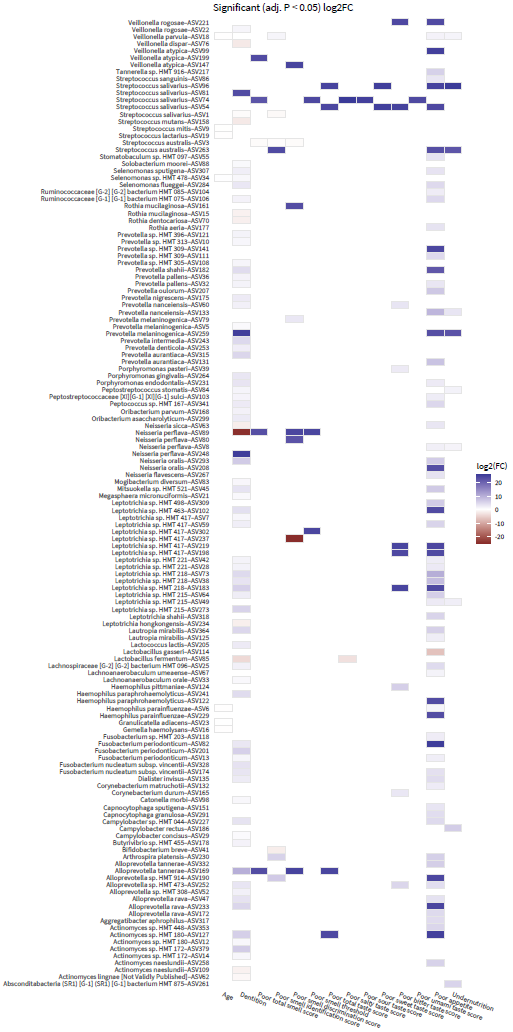


**Supplementary Figure S1: Microbiota taxa univariately associated with age, dentition, poor taste, smell, appetite, and undernutrition outcomes**

Heatmap depicting log2 fold change for species that are significantly (Benjamini-Hochberg adjusted p-value <0.05) associated with age, dentition, all poor smell scores, all poor taste scores, poor appetite, and undernutrition. Blue indicates species that increase in abundance with these conditions and red indicates species that decrease in abundance.

| **Supplementary Table S6: Proportion of participants with no *Candida albicans*/low *Candida albicans* abundance/high *Candida albicans* abundance that have poor taste/smell/appetite or undernutrition** | | | | |
| --- | --- | --- | --- | --- |
|  |  | **No *C. albicans*** | **Low *C. albicans*** | **High *C. albicans*** |
|  | **n** | **n=192** | **n=82** | **n=82** |
| Poor total taste  Normal total taste | 355 | 16 (8.4)  175 (91.6) | 7 (8.5)  75 (91.5) | 10 (12.2)  72 (87.8) |
| Poor sweet taste  Normal sweet taste | 355 | 27 (14.1)  164 (85.9) | 15 (18.3)  67 (81.7) | 13 (15.9)  69 (84.1) |
| Poor sour taste  Normal sour taste | 355 | 60 (31.4)  131 (68.6) | 26 (31.7)  56 (68.3) | 26 (31.7)  56 (68.3) |
| Poor salty taste  Normal salty taste | 355 | 65 (34.0)  126 (66.0) | 32 (39.0)  50 (61.0) | 37 (45.1)  45 (54.9) |
| Poor bitter taste  Normal bitter taste | 355 | 12 (6.3)  179 (93.7) | 6 (7.3)  76 (92.7) | 8 (9.8)  74 (90.2) |
| Poor umami taste  Normal umami taste | 355 | 61 (31.9)  130 (68.1) | 25 (30.5)  57 (69.5) | 29 (35.4)  53 (64.6) |
| Poor total smell  Normal total smell | 139 | 10 (16.9)  49 (83.1) | 4 (14.8)  23 (85.2) | 9 (17.0)  44 (83.0) |
| Poor smell threshold  Normal smell threshold | 141 | 16 (26.7)  44 (73.3) | 7 (25.0)  21 (75.0) | 8 (15.1)  45 (84.9) |
| Poor smell discrimination  Normal smell discrimination | 142 | 5 (8.1)  57 (91.9) | 5 (8.1)  22 (81.5) | 18 (34.0)  35 (66.0) |
| Poor smell identification  Normal smell identification | 355 | 26 (13.6)  165 (86.4) | 17 (20.7)  65 (79.3) | 21 (25.6)  61 (74.4) |
| Poor appetite  Normal appetite | 355 | 11 (5.8)  180 (94.2) | 1 (1.2)  81 (98.8) | 8 (9.8)  74 (90.2) |
| Undernutrition  No undernutrition | 356 | 40 (20.8)  152 (79.2) | 15 (18.3)  67 (81.7) | 21 (25.6)  61 (74.4) |
| Shown are number (percentages). | | | | |
| Poor taste: total taste score < 6, sweet score < 2, sour score < 2, salty score < 2, bitter score < 1, umami score < 1. | | | | |
| Poor smell: TDI-score ≤19.5, T-score ≤2.5, D-score ≤ 7, I-score ≤ 9. | | | | |
| Poor appetite: Council of Nutrition Appetite Questionnaire score <28. | | | | |
| Undernutrition: >5% bodyweight loss averaged over 2 years or BMI<20 (if age <70) or BMI<22 (if age >70). | | | | |

| **Supplementary Table S7: qPCR primers** | | | | | |
| --- | --- | --- | --- | --- | --- |
| **name** | **Target species** | **gene target** | **5'-3' sequence** | **label** | **quencher** |
| CaF^1^ | Candida albicans | 18S | GGG TTT GCT TGA AAG ACG GTA |  |  |
| CaR^1^ | Candida albicans | 18S | TTG AAG ATA TAC GTG GTG GAC GTT A |  |  |
| CaP^1^ | Candida albicans | 18S | ACCTAAGCCATTGTCAAAGCGATCCCG | FAM | TAMRA |
| ^1^Guiver M, Levi K, Oppenheim BA. Rapid identification of candida species by TaqMan PCR. J Clin Pathol. 2001;54(5):362-6. | | | | | |
| . | | | | | |
